# Inflammatory Pseudotumor Formation at a Port Site after Robotic Partial Nephrectomy for Renal Cell Carcinoma

**DOI:** 10.1155/2020/8884409

**Published:** 2020-08-15

**Authors:** Mitsuo Ofude, Ryunosuke Nakagawa, Satoko Urata, Tohru Miyagi, Takao Nakashima, Hiroshi Minato

**Affiliations:** ^1^Department of Urology, Ishikawa Prefectural Central Hospital, 2-1 Kuratsukihigashi, Kanazawa, Ishikawa, Japan; ^2^Department of Diagnostic Pathology, Ishikawa Prefectural Central Hospital, 2-1 Kuratsukihigashi, Kanazawa, Ishikawa, Japan

## Abstract

Inflammatory pseudotumors (IPTs) are benign masses arising from nonspecific inflammatory conditions including surgical invasion. We herein report the rare case of an IPT mimicking port-site metastasis in a 69-year-old patient who underwent retroperitoneal robotic partial nephrectomy for stage T1a renal cell carcinoma. Radiological examination performed six months after the surgery revealed the presence of a mass underneath the abdominal wall which coincided with a port site. The tumor was resected by laparoscopic transperitoneal approach, and histological examination led to the diagnosis of an IPT that consists of xanthogranulomatous inflammation. We also discuss the etiology of IPT formation and features distinguishing IPTs from port-site metastasis.

## 1. Introduction

Inflammatory pseudotumors (IPTs) which are caused by nonspecific inflammatory conditions can develop at nearly every body site [1]. Albeit the very low frequency, several cases of patients who developed IPTs after surgery have been reported even in the era of minimally invasive surgery [2–6]. Distinguishing IPTs from local recurrence or metastasis of malignant neoplasms can be challenging. We herein present the rare case of an IPT mimicking port-site metastasis (PSM) of renal cell carcinoma (RCC) after robotic partial nephrectomy (RPN).

## 2. Case Presentation

**Figure 1:**
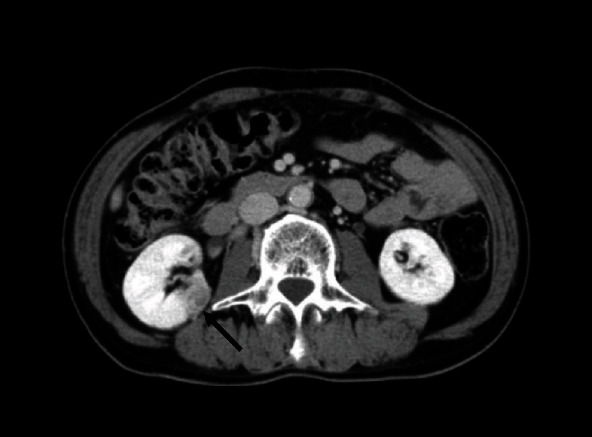
Contrast-enhanced computed tomography shows a right renal tumor (arrow) with a maximum diameter of 27 mm.

A 69-year-old Japanese male with diabetes mellitus underwent right-sided retroperitoneal RPN for an incidental renal tumor (Figure 1). Five ports including a 12 mm AirSeal™ assistant port were placed in the procedure. The tumor resection was performed under warm ischemia, and the renal parenchymal defects were repaired using a continuous 2-0 V-Loc™ suture over a Surgicel™ bolster. The resected specimen was extracted using an entrapment bag. A drainage tube was left in a robotic instrument port. Port sites were closed with one stitch using 0 Vicryl™. Histological examination led to the diagnosis of grade 2, stage pT1a clear-cell RCC with negative surgical margins. No adjuvant therapy was administered.

**Figure 2:**
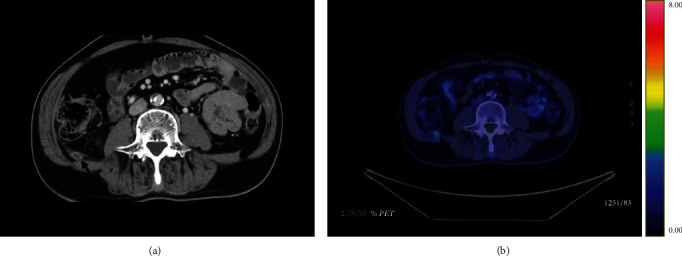
Appearance of a mass underneath in right side of the abdominal wall (arrow). (a) Contrast-enhanced computed tomography. (b) [18F]fluorodeoxyglucose- (FDG-) positron emission tomography-computed tomography shows increased FDG uptake with a maximum standardized uptake value of 3.2.

Follow-up computed tomography (CT) scan performed six months after the surgery revealed an emerging small mass below the right side of the abdominal wall coinciding with a 12 mm AirSeal™ assistant port site (Figure 2(a)). By [^18^F]fluorodeoxyglucose- (FDG-) positron emission tomography- (PET-) CT, the mass had a mild FDG uptake with a maximum standardized uptake value of 3.2 (Figure 2(b)). Further evaluation did not reveal elevated levels of inflammatory markers in blood. Overall, these findings were compatible with PSM of RCC.

**Figure 3:**
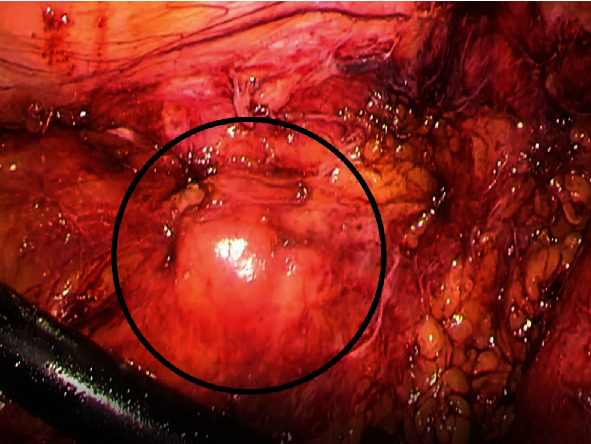
Intraoperative image reveals a mass (circle) observed in the peritoneal cavity.

**Figure 4:**
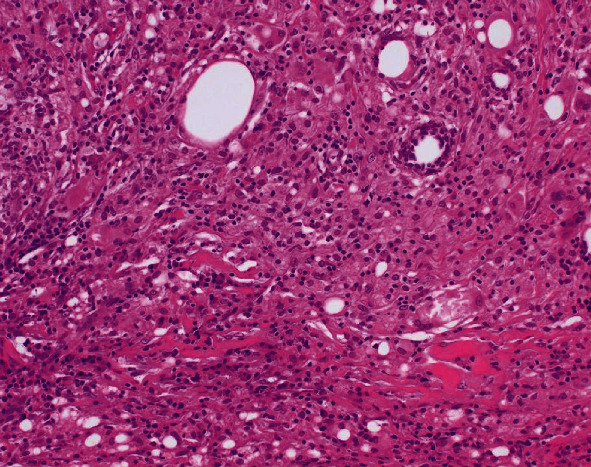
Histology of the mass at a port site showing aggregation of foamy histiocytes with lipogranulomas, mild infiltration of lymphocytes and plasma cells, and focal fibrosis.

Tumor resection was subsequently performed by laparoscopic transperitoneal approach (Figure 3). Although peeling the tumor off the abdominal wall was difficult due to strong adhesion, en bloc resection was successful. The resected specimen included a yellow mass, 25 × 20 × 13 mm in size, without foreign bodies. Histological examination revealed that the mass was composed of aggregation of foamy histiocytes with fat necrosis (FN), lipogranulomas, fibrosis, and mild infiltration of lymphocytes and plasma cells (Figure 4). The pathological diagnosis was an IPT that consists of xanthogranulomatous inflammation. The postoperative course was uneventful, and no evidence of recurrence was observed six months after the IPT resection.

## 3. Discussion

IPTs are benign tumors which mimic malignant neoplasms and comprise cells associated with both acute and chronic inflammation. The pathogenesis and etiology of IPTs are unspecific; therefore, IPTs have been described under various names such as xanthogranuloma, inflammatory myofibroblastic tumor [1], and lipogranuloma [4] based on histological findings or cellulose granuloma [2], surgical granuloma [3], and Schloffer's tumor described as an immune response to foreign bodies used in surgery [5, 7] according to the antigens provoking inflammation.

Schloffer's tumor might be considered the diagnosis in the present case based on the potential etiology because it is possible that Vicryl™ suture or Surgicel™ used in RPN might have provoked a foreign body reaction. Asano et al. [5] reported four patients who developed Schloffer's tumors after colorectal surgery; absorbable sutures were used in prior surgeries in all four cases. The time interval was less than one year between the prior surgery and tumor diagnosis in three of these cases. The shared preoperative clinical findings between these cases and the present case include positive FDG-PET uptake in the absence of elevated inflammatory marker levels in peripheral blood. However, the tumor in the present case was located underneath the abdominal wall and not inside the abdominal cavity, and no tumors developed at other port sites where Vicryl™ sutures were used. These findings overall suggest that Vicryl™ sutures were not likely to have provoked a foreign body reaction.

In the present case, the Surgicel™ bolster placed on the renal parenchymal defects was nearly invisible at the IPT site. This image finding shows that the Surgicel™ bolster was normally diminished with time after surgery [8]. It is unlikely that a deviating Surgicel™ fragment provoked a foreign body reaction in the present case.

While the exact cause of IPT in the present case remains unknown, we speculate that the following two processes were more likely etiologies. The first one is chronic inflammation by microabscess formation underneath the abdominal wall, which might be partially due to diabetes mellitus. The second is the involvement of FN. Fat fragments produced in RPN might migrate to a port site and subsequently develop FN provoking chronic inflammation. Amblee and Ganesh [6] reported an interesting case of IPT formation caused by FN in right-sided upper perinephric fat after left adrenalectomy for Cushing's disease. However, the exact location of IPT seems to be unclear because tumor excision had not been performed in their case. We consider that isolated fat fragments in left adrenalectomy might lead to FN in right-sided abdominal location. We do not routinely irrigate the operative field after RPN, but careful irrigation and suction could prevent form abscess or remaining fat fragments which causes IPT formation.

The preoperative differentiation between IPT and PSM of RCC is difficult because of the rarity of both conditions, which were reported in very few case reports [2–4, 9–11]. Based on the limited information, both IPT and PSM of RCC are usually asymptomatic while retaining a small tumor burden. The diagnosis depends primarily on imaging studies; however, there are no distinguishable radiologic features of IPT, which vary according to the histological content and location [1]. Additionally, FDG-PET appears to be impractical as both conditions can display positive FDG-PET uptake [4, 5, 10].

Song et al. [11], who reported 16 cases of PSM after laparoscopic and robotic surgery for RCC, found that PSM development was associated with surgical technical approaches such as specimen morcellation before extraction, lack of entrapment bags during extraction, and tumor rupture in seven cases; the authors reported that the remaining nine cases had uniformly aggressive initial RCC of grade 3 or higher. In comparison, the risk of PSM might have been lower in the present case. However, Shimokihara et al. [10] reported a case of PSM with an initially low-risk RCC similar to the present case. Therefore, we suggest that any tumor suspicious for PSM should be resected.
